# Therapeutic potential of flavonoids in erosive tooth wear management: a scoping review

**DOI:** 10.1007/s00784-025-06623-8

**Published:** 2025-10-27

**Authors:** Gabriel Pereira Nunes, Renata de Oliveira Alves, Geórgia Rondó Peres, Priscila Toninatto Alves de Toledo, Aline Rogéria Freire de Castilho

**Affiliations:** 1 Department of Prosthodontics and Periodontology, Piracicaba Dental School, University of Campinas (UNICAMP), Piracicaba, SP Brazil; 2 Department of Preventive and Restorative Dentistry, Araçatuba School of Dentistry, São Paulo State University (UNESP), Araçatuba, SP Brazil; 3 Department of Operative Dentistry, Endodontics and Dental Materials, Bauru School of Dentistry, University of São Paulo (USP), Bauru, SP Brazil; 4 Department of Pediatric Dentistry, Indiana University School of Dentistry, 415 Lansing St, OH145, Indianapolis, IN 46202 USA

**Keywords:** Flavonoids, Erosive tooth wear, Dental enamel, Dentin

## Abstract

**Objectives:**

This scoping review provides an overview of the use of flavonoids in managing erosive tooth wear, including preventive methods, therapeutic interventions, and strategies for its control.

**Materials and methods:**

This scoping review was conducted in accordance with the PRISMA-ScR statement. A comprehensive search was conducted across multiple databases, retrieving all available records up to January 2025. Eligible evidence included in vivo, in vitro, and in situ studies investigating the therapeutic potential of flavonoids on erosive tooth wear. Data were extracted, and synthesis of study findings was performed.

**Results:**

Of the 391 records screened, 34 studies were included in the review: two randomized clinical trials, 20 in vitro studies, five in situ investigations, and seven studies employing both in vitro and in *situ* approaches. Flavonoids demonstrated promising potential in the control of erosive tooth wear. Epigallocatechin-3-gallate (EGCG) was the most extensively studied and proved more effective than fluoride and chlorhexidine in dentin preservation, tissue loss reduction, and long-term adhesive performance. Proanthocyanidin also proved effective, especially in collagen stabilization and improved bond strength to eroded dentin. Quercetin stood out for its protective and reparative effects, including superior tubular occlusion, mechanical reinforcement, and preservation of the organic matrix. Although less investigated, theaflavin and hesperidin displayed dose-dependent effects in reducing wear and inhibiting matrix metalloproteinase activity.

**Conclusions:**

Flavonoids, especially EGCG, proanthocyanidin, and quercetin, show promising results in controlling erosive tooth wear, with multiple mechanisms including remineralization, anti-collagenolytic activity, and biomodification of dentin.

**Clinical relevance:**

Flavonoids may act as valuable adjunctive agents in preventive and restorative strategies for erosive tooth wear. However, further clinical trials are required to confirm their efficacy and establish optimal formulations for clinical application.

**Supplementary Information:**

The online version contains supplementary material available at 10.1007/s00784-025-06623-8.

## Introduction

Throughout life, teeth are continuously exposed to various physical, mechanical and chemical agents, whose effects, at different intensities, contribute to the wear of hard dental tissues [1–3]. This wear, characterized by its irreversible nature, can occur as part of the natural aging process or be exacerbated by lifestyle factors [4], with a prevalence reaching up to 61.6% [5]. Tooth wear refers to the progressive loss of mineralized dental tissues, resulting from processes such as erosion, attrition, abfraction, and abrasion [6]. Among these conditions, erosive tooth wear is particularly prevalent, with global estimates indicating a prevalence of 30% to 50% in primary teeth and 20% to 45% in permanent teeth [7]. Additionally, due to lifestyle and behavioral factors, adolescents represent a significant at-risk group, exhibiting a high prevalence of this condition in this age group [8]. An example of this is the increased consumption of acidic foods and beverages, as well as factors such as gastroesophageal reflux, which are frequently associated with erosive dental wear [1, 5].

Given the high prevalence and multifactorial nature of tooth wear, mainly erosive tooth wear, preventive strategies remain the most effective approach to minimizing its impact on mineralized dental tissues [9, 10]. However, once the process is initiated, often silently and progressively, therapeutic interventions become necessary to halt its progression, which may be accelerated by various intrinsic and extrinsic factors [2, 11]. Although there is no consensus in the literature regarding the most appropriate treatment, bioactive agents, especially topically applied fluoride-based compounds, have been widely recommended in the clinical management of this condition [10–12]. Fluoride acts on the demineralization and remineralization processes of dental tissues by reducing hydroxyapatite dissolution and enhancing resistance of the tooth structure to acidic challenges [12, 13]. However, its effectiveness depends on the presence of fluoride in a free and soluble form within the saliva or acquired pellicle, which may limit its therapeutic potential under frequent acid exposure [12, 14].

In light of these limitations, other agents such as calcium phosphate compounds, chlorhexidine (CHX), potassium nitrate, chitosan, and titanium tetrafluoride (TiF_4_) have been explored mainly for their potential in managing erosive tooth wear and enhancing restorative outcomes [15–17]. Their use is particularly relevant in cases of erosive tooth wear involving dentin exposure, where the degradation of the demineralized organic matrix by matrix metalloproteinases (MMPs) plays a central role in tissue breakdown [18]. While MMPs substantially contribute to the progression of erosive lesions, they are not the sole factor driving tissue loss [19]. Repeated and short-term episodes of low pH, such as those caused by the frequent intake of acidic fruit juices [19, 20], directly trigger enamel and dentin demineralization. Moreover, the limited buffering capacity of saliva to neutralize these fluctuations exacerbates the erosive challenge, amplifying overall tissue damage [21]. In this context, these substances exhibit antiproteolytic properties capable of inhibiting MMP activity, thereby preserving the structural integrity of the dentin matrix and promoting its biomodification [22]. Additionally, by enhancing dentinal tubule occlusion and improving long-term adhesive system performance, these agents are also investigated in the context of restorative procedures [15]. It is worth noting that they are frequently used as control or comparator treatments in studies evaluating novel therapeutic agents. However, despite their benefits, current evidence indicates that these strategies do not provide complete protection and may be insufficient to fully prevent the progression of erosive tooth wear [14, 23].

This scenario has increased interest in alternative strategies to enhance the protective effects against erosive tooth wear. Among them, natural compounds derived from plants, especially flavonoids, have shown promising results [24–26]. Flavonoids are polyphenolic compounds naturally present in fruits, vegetables, and other plant sources [27]. They have demonstrated beneficial effects in the treatment of several oral conditions, including mucositis, candidiasis, periodontal disease, endodontic infections, and apical periodontitis [28–33]. Their anti-inflammatory, antimicrobial, and antioxidant properties contribute to these beneficial effects, making them attractive candidates in oral therapeutics [27, 34].

Importantly, flavonoids have also shown potential in protecting and treating alterations in mineralized dental tissues, such as dental caries and erosive tooth wear [22, 24, 29, 35, 36]. Studies indicate that flavonoids can enhance enamel and dentin resistance to acid-induced demineralization by modulating mineral loss and stimulating remineralization [25, 26, 37]. In addition, their potent antioxidant capacity allows them to neutralize reactive oxygen species and inhibit enzymes such as MMPs, which are involved in oxidative tissue damage, mechanisms that are key to limiting the progression of erosive wear [25, 38]. Despite there are reviews on the effects of flavonoids in various oral conditions [29, 38, 39] to date no scoping review has comprehensively consolidated evidence on their role in preventing and/or treating erosive tooth wear. Therefore, this review provides an overview of the use of flavonoids in managing erosive tooth wear, including preventive methods, therapeutic interventions, and strategies for its control.

## Materials and methods

### Study protocol

This scoping review was conducted in accordance with the PRISMA-ScR (Preferred Reporting Items for Systematic Reviews and Meta-Analyses extension for Scoping Reviews) guidelines [40], and the methodological approach was guided by recently published studies [38, 41].

### Eligibility criteria

The research question was formulated based on the Patient, Concept, and Context (PCC) framework, resulting in the following inquiry: “Is the use of flavonoids effective as a preventive or therapeutic approach for managing tooth wear?”

- *Population*: Studies involving human participants undergoing preventive, restorative, or therapeutic dental interventions for tooth wear, as well as in vitro or in situ studies evaluating the effects of flavonoids on mineralized dental tissues, such as enamel and dentin.
- *Concept*: Use of flavonoids in any formulation, such as treatment solutions, incorporation into dental biomaterials, or surface coatings, aimed at promoting protective or reparative effects on dental hard tissues. The comparison groups included either a negative control (e.g., placebo or no treatment) or a positive control involving active agents routinely used in dental practice for similar purposes, such as fluoride, chlorhexidine, or other bioactive compounds employed in the management of tooth wear.
- *Context*: Outcomes included alterations in mineralized dental structures, such as wear tooth, changes in mineral content, surface topography, microhardness, resistance to acid challenges, demineralization and remineralization rates, adhesive performance, and structural or compositional changes in enamel and dentin. Additionally, effects on the organic matrix of dentin were considered. These outcome measures aim to evaluate the preventive effects on erosive tooth wear, as well as restorative interventions targeting mineralized dental tissues, in both clinical and *in vitro/in situ* settings.

The inclusion criteria for this scoping review encompassed clinical trials, in situ, and in vitro studies that investigated the effects of flavonoids on tooth wear. Eligible studies were those that applied flavonoids in solution or in dental materials aimed at preventing or treating dental erosion. To ensure clarity of the intervention’s impact, studies were included only if the presence of flavonoids was the sole variable differing between experimental groups. Studies were excluded if they lacked a control group, evaluated multiple agents without isolating the specific effect of flavonoids, or used plant extracts without isolating the flavonoid of interest. In addition, reviews and expert opinions were not considered. Overall, any research failing to meet these predefined criteria was excluded. There were no restrictions on publication date or language, allowing for a comprehensive exploration of the topic.

### Search strategy

The electronic literature search was conducted across multiple comprehensive databases, including PubMed (MEDLINE), Scopus, Web of Science, Embase, and the Cochrane Library, covering publications up to January 10, 2025. Gray literature, including outputs from government, academic, business, and industrial sectors not controlled by commercial publishers, was also explored via the OpenGrey database (http://www.opengrey.eu/). To maximize the search sensitivity, Medical Subject Headings (MeSH) terms were carefully selected in collaboration with a specialized librarian and supplemented with free-text keywords not indexed as database descriptors (see Appendix #S1). In addition to electronic searches, a manual review was performed to identify any potentially missed manuscripts. Additionally, reference lists of all included studies were screened to uncover further relevant articles that might have been overlooked.

### Study selection

The article selection process involved a systematic two-step approach. Initially, titles and abstracts were screened to identify potentially eligible studies, followed by a thorough evaluation of their full texts. Two independent reviewers (GRP and ROA) conducted the initial screening, importing all references retrieved from the databases into an online reference manager (EndNote Web; Thomson Reuters Inc., Philadelphia, PA, USA) to remove duplicates. When titles and abstracts lacked sufficient information, the full texts of the studies were retrieved for detailed analysis. Any disagreements during this phase were resolved through discussion or consultation with a third reviewer (GPN).

In the subsequent step, after duplicates were excluded, articles deemed potentially relevant underwent a screening based on their titles and abstracts. Studies meeting the inclusion criteria were then subjected to a comprehensive full-text review, with reasons for exclusion carefully documented. The final selection of studies was independently performed by the two reviewers, with any conflicts resolved by the third reviewer to ensure consensus. Agreement between the two reviewers on title and abstract screening was assessed using Cohen’s kappa coefficient (κ).

### Data extraction and synthesis

Study details were extracted using customized data collection forms designed to capture comprehensive information, including authorship, publication year, country of origin, study design, targeted dental tissue, flavonoid and control agents, mode of administration or application, assessment methods, main outcomes, and intervention effects. The specific types of flavonoids investigated were systematically documented (Table 1). When necessary, corresponding authors were contacted via email to obtain additional clarifications.

**Table 1:** General data from included studies

Author/year (Country)	Design study	Dental tissue	Flavonoid (s)	Control (s)	Approach, form of administration, and protocol	Assessment method	Main results	Grade of the treatment effectiveness*
Alves et al., 2023 (Brazil)	Randomized Clinical Trial	Dentin (Abfraction) Human premolars teeth 30 samples/per group	EGCG: 0.02%	Placebo - Distilled water	Treatment approach – Solution. EGCG was applied to dentin with a microbrush for 1 min, followed by a 5 s air jet and excess removal with absorbent paper. The control group received distilled water. Two adhesive layers were applied for 10s.	Clinical and photographic criteria	EGCG presented effects similar to the control, with abfraction lesions not significantly influencing the survival of the restorations.	(=)
Chen et al., 2012 (China)	Randomized Clinical Trial	Enamel/Dentin (Eroded) 32 participants per group	EGCG: 1% and 2%	Placebo gel	Treatment approach – Gels. 1 mL of gel was dispensed into the custom trays, targeting the regions corresponding to the anterior teeth of both dental arches. Participants were instructed to wear the trays overnight during sleep (approximately 8 h) and to remove them the following morning.	Content of ICTP	EGCG group, ICTP concentrations decreased significantly over time, stabilizing in the third week, while in the control group there was a progressive increase.	(+)
Martins et al., 2024 (Brazil)	In situ	Enamel (Erosion/erosion and abrasion) Bovine teeth 42 samples/per group	Proanthocyanidin: 2%	Placebo - Deionized water; SnCl2/NaF/AmF	Preventive approach – Solution. Volunteers formed enamel pellicles in two daily 30 min sessions. After treatments, the appliance was reinserted for 1 h. In vitro, blocks underwent 5 days of 4 daily erosive cycles (90s in 0.5% citric acid) and 15 s abrasive cycles after the first and third erosive cycles, with 2 h saliva immersion between cycles.	Profilometry	Proanthocyanidin showed a protective effect, with the proanthocyanidin group exhibiting an enamel wear rate similar to the positive control.	(+)
Bueno et al., 2022 (Brazil)	In situ	Dentin (Erosion + Abrasion) Bovine teeth 8 samples/per group	Proanthocyanidin (PA): 10% PA 10% + NaF 1100 ppm	Placebo; CHX: 0.012%; NaF: 1100 ppm	Treatment approach – Toothpaste. Ten volunteers wore palatal appliances with dentin specimens, each phase lasting 5 days with a 7-day interval. Erosive challenges involved Coca-Cola immersion (5 min, 3x/day, 5 days). Abrasive challenges followed the 1 st and 3rd erosive cycles using an electric toothbrush (30 s with toothpaste slurry). Appliances were worn 12 h/day and stored in humid containers overnight.	Profilometry	Proanthocyanidin and the combination of proanthocyanidin and fluoride dentifrices revealed the best results, showing that these formulations could be a promising alternative for patients who suffer with dentin erosion.	(+)
Hong et al., 2022 (China)	In situ In vitro	Dentin (Erosion and abrasion) Human third molars teeth 10 samples/per group	Quercetin: 75 µg/mL 150 µg/mL 300 µg/mL	Placebo - Deionized water; Ethanol CHX: 120 µg/mL	Preventive approach – Solution. Treatment for 2 min and then subjected to in situ/in vivo erosive/abrasive challenge for 7 days follows: in vivo erosion 4 times a day and then in vivo toothbrush abrasion after the first and last erosive challenges of each day.	Dentin loss, inhibition of dentin-derived MMPs, degrees of crosslinking, and ultimate microtensile	Quercetin reduced dentin loss, mainly at higher concentrations, despite limited penetration to superficial dentin layers. Increased concentrations enhanced collagen crosslinking, resulting in improved mechanical strength.	(+)
DE Moraes et al. 2022 (Brazil)	In situ In vitro	Dentin (erosion) Human Teeth (In vitro) 3 samples/per group 20 voluntaries (In situ)	EGCG: 0.1% and 0.0014%	Negative control (no treatment)	Treatment approach – Solution. The subjects wore upper removable devices containing four dentin blocks. Erosive challenge (coke-1 min) was performed four times/day/5 days. Blocks were treated for 1 min.	Microhardness analysis	Regarding differences observed in dentin roughness or wear there was no significant difference between the treatments.	(=)
Siqueira et al., 2021 (Brazil)	In situ	Dentin (erosion) Human teeth 8 sample/per group	Proanthocyanidin: 6.5%	Placebo	Treatment approach – Incorporated into the primer - adhesive. Specimens were immersed in a Coca-Cola (pH 2.6) 4x/day for 90 s each (10 ml/specimen) for 5 days. The specimens were then rinsed in deionized water (10 s) and immersed in a remineralizing solution (pH 6.7, 10 ml/specimen) for 1 h between erosive demineralization. The remineralization solution was replaced daily.	Micro-Raman analysis, SEM, Silver nitrate penetration analysis	Proanthocyanidins reduced nanoleakage to levels similar to sound dentin. DMSO increased the degree of conversion in eroded dentin and decreased nanoleakage, regardless of combination with other agents.	(+)
Kato et al., 2021 (Brazil)	In situ	Dentin (erosion/erosion + abrasion) Bovine teeth 40 sample/per group	EGCG: 400 µM	Placebo; CHX: 0.012%	Treatment approach – Gels. For the ERO, appliances were immersed in a cola drink for 5 min, 4x/day, while for ERO + ABR, the blocks were brushed for 15 s with a dentifrice slurry after the 2nd and 3rd erosive challenges. 13 volunteers participated in study 2, in which the treatment was performed only once (1 min) with gels.	Profilometry	EGCG and CHX gels reduced dentin loss compared to placebo. Abrasion did not significantly increase wear over erosion alone for any gel after 5 and 10 days, except in the untreated group, which showed significantly higher wear with combined erosion and abrasion.	(+)
Boteon et al., 2020 (Brazil)	In situ In vitro	Enamel(erosion) Bovine teeth 8 sample/per group	Proanthocyanidin: 6.5% gel	No intervention (neither gel nor acquired pellicle)	Preventive approach – Gels. Gels were applied for 1 min. Then, enamel blocks were immersed in 0.5% citric acid, pH 2.5, for 30 s to promote a short erosive challenge	Microhardness analysis	Acquired pellicle and 6.5% PA gel showed the lowest hardness loss. The control group and Proanthocyanidin gel 6.5% showed no statistically significant difference among them.	(+)
Cardoso et al., 2020 (Brazil)	In situ	Dentin (erosion) Incisive Bovine 16 sample/per group	Proanthocyanidin (PA): 10%; pH 7 and pH 3	Placebo CHX: 0.012%	Treatment approach – Solution. 1 st Phase − 10% PA mouthrinse (pH 7.0), 10% PA mouthrinse (pH 3.0) 2nd Phase − 0.12% CHX mouthrinse), and previous treatment. Each volunteer’s device was subjected was subjected to 3 erosive cycles (5 min) per day for 5 days. Treatments were applied once after the 2nd erosive challenge (5 min).	Microhardness analysis, Optical profilometry	10% PA mouthrinse (pH 7.0) and CHX 0.012% showed significantly lower wear values, but with no statistical difference between them.	(=)
Jiang et al., 2020 (China)	In situ In vitro	Dentin (Eroded) Human third molars 12 samples/per group	Quercetin: 75 µg;150 µg 300 µg/mL; EGCG: 183.2 µg ml	Placebo Deionized water and Ethanol; CHX: 120 µg mL; NaF: 1.23 × 10^4^ µg mL	Treatment approach – Solution. In vitro: Specimens (after the formation of an acquired pellicle) were treated for 2 min and erosive challenges. 7-day erosion cycling regimen (4 erosive challenges daily). In situ: Specimens were treated for 2 min. Then the appliances were worn for 2 h, followed by an erosive challenge.	Surface microhardness, erosive dentin wear, and demineralized organic matrix, and ICTP.	Flavonoid-treated groups, especially with quercetin 300 µg/mL (Q300), exhibited a significant reduction in dentin erosive wear, increased thickness of the DOM, and lower mineral loss, all without affecting color.	(+)
Zimmermann et al.,2019 (Germany)	In situ In vitro	Enamel (Sound) Bovine Teeth	EGCG: 100 and 460 µg/mL	Negative control: Deionized water; Lysozyme 100 µg/mL; 1430 µg/mL; Iron III Chloride (FeCl_3_ ⋅ 6 H_2_O) 27 and 270 µg/mL	Preventive approach – Solution. In vitro pellicle preparation: Cell-free saliva was obtained by centrifuging freshly collected saliva at 4 °C for 15 min. The supernatant was then carefully pipetted onto the substrates and incubated at 37 °C for 90 min. Supernatant was injected into the measuring cell of a quartz crystal microbalance with dissipation monitoring until equilibrium was reached. Following, the samples were rinsed with the astringent solutions used in the experiment.	Measuring cell of a quartz crystal microbalance with dissipation monitoring, Streaming current, ATM, TEM	EGCG concentration-dependently increased the thickness and roughness of the salivary film, which may intensify the sensation of oral astringency. Among the groups analyzed, lysozyme showed the highest astringent potential compared to controls.	(+)
Souza-e-Silva et al., 2017 (Brazil)	In situ	Acquired enamel pellicle Human teeth	EGCG: 400 µg/m	Positive control: CHX: 0.12%	Preventive approach – Gels. The volunteers underwent dental prophylaxis to eliminate the existing acquired enamel pellicle. Following this, a new pellicle was allowed to form over a period of 2 h. Once the pellicle was established, one of the gels was carefully applied to the tooth surfaces using a cotton swab for 1 min, after which the excess gel was removed with a clean swab. Subsequently, an additional h was allotted for further pellicle formation before sample collection.	Quantitative proteomic analysis	EGCG and CHX gels enhanced the presence of protective proteins in the acquired enamel pellicle, including PRPs, calcium-binding proteins, and statherin. Following citric acid challenge, levels of cystatin and profilin-1 increased. Both treatments significantly modified the pellicle’s proteomic profile.	(+)
Kato et al., 2010 (Brazil)	In situ In vitro	Dentin (Sound) Bovine teeth 10 samples per group	EGCG: 10 µM and 400 µM	Placebo; NaF: 1.23%; CHX: 0.012%	Preventive approach – Gels. During the intraoral phase, a thin layer of gel was applied using a microbrush for 1 min, followed by gentle removal with a cotton swab to ensure precision and care.	Contact profilometry, SEM	Gels with MMP inhibitors (EGCG and CHX) fully prevented dentin wear, outperforming NaF and placebo. No wear interface was observed under SEM in MMP groups, and tubular occlusion was evident—unlike in the placebo group.	(+)
Chen et al., 2024 (China)	In vitro	Dentin (Erosion and abrasion) Human third molars teeth 26 samples/per group	Quercetin: 300 µg/mL	Placebo - Deionized water; NaF: 12.3 mg/mL	Treatment approach – Solution. Samples underwent 7 days of erosive (4 cycles/day) and abrasive (2 cycles/day) challenges. Each erosion cycle included 1 h in artificial saliva, 5 min in citric acid (pH 2.45), a 10 s water rinse, and 2 h in renewed saliva. Brushing was performed after the first and final daily cycles. After 7 days, samples were divided into groups, and immersed for 2 min/day, kept in artificial saliva for 2 h, and then subjected to further erosive and abrasive cycles.	Surface profile measurements, CLSM (dentine tubule permeability), ICTP, and SEM	Quercetin demonstrated protective capacity, with obliterated tubules, lower collagen degradation, and reduced erosive dentin loss compared to the control groups.	(+)
Abreu et al., 2023 (Brazil)	In vitro	Dentin (Erosion and Abrasion) Bovine teeth 10 samples/per group	EGCG: 0.5%	Sound dentin and Eroded/abraded dentin (Control groups)	Treatment approach – Toothpaste. Dentin specimens underwent three days of five daily cycles: three erosive (0.1% citric acid, 10 s) and two abrasives (three 15 s brushing sessions after the 1 st and 3rd erosive cycles). Biomodification treatment was applied for 1 min, and specimens were stored in humidity for 24 h post-restoration.	Microtensile bond strength, SEM, and dentin permeability	EGCG presented a protective and preventive effect due to the application of EGCG as a biomodifier, which has shown promising results in enhancing bond strength on both sound and eroded-abraded dentin substrates.	(+)
Cardoso et al., 2023 (Brazil)	In vitro	Dentin (Eroded) Human molar teeth 10 samples/per group	Proanthocyanidin: 10%	Placebo; CHX: 0.12%; NaF: 1.23%	Treatment approach – Gels. Dentin specimens were demineralized by immersion in 0.87 M citric acid (36 h at 4 °C). The samples were then thoroughly rinsed in deionized water (30 s). Excess water was removed with absorbent paper.	Contact profilometry, ICTP, and ELISA	Proanthocyanidin showed a protective effect, reducing tooth wear and decreasing DOM degradation compared to the control groups.	(+)
Guo et al., 2023 (China)	In vitro	Dentin (Eroded) Human molars teeth 5 samples/per group	Theaflavins: 1, 2, 4, and 8% EGCG: 1%	Ethanol: 10%; CHX: 1%	Treatment approach – Solution. Dentin blocks underwent 7 days of erosion cycling. After drying, they were treated with a solution for 30 s, then immersed in artificial saliva for 2vh. Each cycle included 5 min in phosphoric acid (pH 2.3, 37 °C), 10 s ultrasonic cleaning, and 1 h in artificial saliva. Four cycles were performed daily, with overnight saliva storage at 37 °C.	CLSM, SEM, zymography, visualization of collagen-ligand complex, microtensile strength and FT-IR.	Theaflavin demonstrated superior efficacy in preventing or slowing erosive dentin wear. Its protective effect is attributed to the inhibition MMPs, collagen crosslinking, and the induction of hydrophilic modifications in dentin collagen.	(+)
Capalbo et al., 2022 (Brazil)	In vitro	Dentin (Erosion) Bovine teeth 12 samples/per group	Quercetin: 0.03%	Placebo; NaF: 0.024%	Preventive approach – Solution. Blocks were treated with 4 mL of each solution twice a day, before the erosive challenges. They were then immersed in citric acid (pH 3.2) for 4 daily erosive challenges (90 s each) over 5 days, with intervals in artificial saliva at 37 °C. After each treatment, the blocks were washed and dried.	Profilometry, Cross-sectional hardness, Zymography, and SEM	The quercetin group exhibited a protective effect, with lower dentin loss, higher integrated dentin hardness in depth, reduced MMP-2 and MMP-9 activity, and obliterated tubules compared to the negative control.	(+)
Iftikhar et al., 2022 (Pakistan)	In vitro	Enamel (sound) Human premolars and impacted third molars 14 samples per group	EGCG	Placebo - Distilled water; Potassium nitrate	Preventive approach – Solution. Tooth specimens were treated in synthetic saliva for 6 h, then immersed in treatment solutions for 4 min. For erosion, samples were immersed in a commercial beverage for 15 min with stirring, rinsed, and placed in artificial saliva for 1 h. This cycle was repeated four times.	SEM and Microhardness analysis	The EGCG group showed lower surface roughness than the water group. However, compared to the KNO_3_ group, there was no statistically significant difference.	(+)
Li et al., 2022 (China)	In vitro	Dentin (erosion) Human third molars teeth 10 sample/per group	Quercetin: 300 µg/ml	Placebo - Deionized water; CHX: 120 µg/ml; NaF: 1.23 × 104 µg/mL	Treatment approach – Solution. Specimens underwent four daily erosive challenges for five days. In one subgroup, specimens were treated with solutions for 2 min before and 5 min cola immersion. In the other subgroup, cola immersion occurred first, followed by a 2 min treatment with the respective solutions.	Optical profilometry, SEM	Quercetin effectively controlling dentin erosion, resulting in significantly lower dentin loss and more pronounced tubule narrowing when compared with the placebo and the NaF group. However, it obtained a similar result to the CHX group.	(+)
Koruyucu et al., 2022 (Turkey)	In vitro	Dentin (Eroded) Human third molars teeth 11 samples per group	EGCG: 0.61%	CHX: 0.2% and 12% NaF: 1100 ppm	Treatment approach – Solution. Samples underwent erosive demineralization by immersion in Coke^®^ (pH 2.6) for 1 min, four times daily, for 5 days. After each immersion, they were rinsed and stored in daily refreshed artificial saliva. Remineralization involved immersion in group solutions for 1 m, four times daily, for 5 days.	Profilometry and SEM	EGCG was found to be as successful as CHX and NAF in terms of transforming demineralized areas into remineralized areas.	(+)
Leal et al., 2021 (Brazil)	In vitro	Dentin (erosion/erosion + abrasion) Human Teeth 10 sample/per group	Hesperidin: 01%, 0.5%, and 1%; EGCG: 0.46%	Placebo: distilled water	Treatment approach – Solution. The specimens were submitted to a cycle (3x/day) for 5 days that consisted of immersion on 1% citric acid (5 min), artificial saliva (60 min), treatment (5 min), brushing (150 movements only in experiment 2), and artificial saliva (60 min/overnight).	Optical profilometry - SEM	EGCG resulted in the lowest tooth wear, similar to water and significantly less than all hesperidin-treated groups, which showed no differences among concentrations. The Water + Col group exhibited the highest wear in both experiments.	(+)
Vertuan et al., 2021 (Brazil)	In vitro	Dentin (erosion) Incisive Bovine 15 sample/per group	Proanthocyanidin (PA): 10%	No treatament CHX: 0.012% TiF4 Varnish	Preventive approach – Gels. 0.012% CHX gel, 10% proanthocyanidin gel or untreated for 1 min; Final Treatment varnish placebo or untreated for 6 h. The samples were submitted to a pH cycling for 5 days: 0.1% citric acid (4 × 90s/day) and artificial saliva between the challenges.	Optical proilometry -SEM	CHX or PA did not affect the progression of EDL. However, the final application of TiF₄ varnish provided significantly better protection against EDL progression than other treatments, independent of the DOM condition and pretreatment used.	(+)
Yu et al., 2021 (China)	In vitro	Dentin Human third molars 32 sample/per group	EGCG: 10%	DMSO	Treatment approach – Solution. Control group: Occlusal surface of the discs was treated by applying sterile deionized water twice with a prophylaxis cup at low speed for 30 s. EGCG group: The occlusal surface of the discs was treated by applying a 2x EGCG suspension according to the powder/liquid ratio of 10 mg/100 µL using a prophy cup at low speed for 30 s.	SEM, Release Ca, and P, and in occlude dentin Ca and P	EGCG effectively occluded dentinal tubules within 1 week and maintained obstruction after 1 month. EGCG release was rapid initially and sustained over 30 days, along with stable Ca and P ion release.	(+)
Zamperini, et al., 2021 (USA)	In vitro	Dentin (erosion) Incisive Bovine 20 sample/per group	EGCG	Placebo; NaF: 1000 ppm	Treatment approach – Solution. Root dentin specimens were subjected to an erosion-remineralization cycling model (6×/day; 5 days) that included 5-min immersion in 1% citric acid and 60-min immersion in remineralization solution. At the remineralization half-time, the specimens were treated with EGCG, or NaF.	Profilometry	Under pH-cycling, EGCG led to greater erosion depth compared to both controls. However, under pH-proteolytic cycling, EGCG significantly reduced tissue loss relative to the negative control, demonstrating its anti-erosive effect on root dentin in the presence of proteolytic activity.	(+)
de Siqueira et al., 2020 (Brazil)	In vitro	Dentin (erosion) Human molars 11 sample/per group	Proanthocyanidin: 6.5%	Placebo	Treatment approach – Incorporated into the primers-adhesive. Surface dentin was immersed for 60 s at 6 h intervals daily for 5 days. After each erosive cycle, the specimens were rinsed with deionized water (10 s) and immersed in a remineralizing solution (1 h).	Microtensile bond strength, nano-hardness, and Young’s modulus measurements	Regarding dentin adhesion, the Proanthocyanidin: 6.5% group, demonstrated a superior result to the placebo group.	(+)
Landmayer et al., 2020 (Brazil)	In vitro	Dentin (Eroded) Human third molars 7 samples per group	Proanthocyanidin: 10%; EGCG: 400 μm;	Placebo; CHX: 0.12%	Treatment approach – Gels. A thin layer of the gels was applied with a microbrush and removed with cotton swabs after passive action for 5 min.	Microtensile bond strength	Gels containing CHX, EGCG, or placebo showed similar effects on the bond strength of the adhesive system to simulated eroded dentin, compared to the untreated control group.	(+)
Costa et al., 2019 (Brazil)	In vitro	Dentin (Sound and eroded) Human third molars 6 samples per group	EGCG: 0.1%	Placebo - Distilled wáter; CHX: 2%	Preventive approach – Solution. Dentin surfaces were pretreated with 15 µl of each solution and scrubbed for 60 s. The excess was removed with absorbent paper. The specimens were stored in distilled water at 37 °C for 24 h.	Microtensile bond strength, Failure Modes	EGCG did not significantly affect bond strength to dentin, maintaining adhesive performance over time, while CHX reduced this strength, especially in eroded dentin.	(+)
Siqueira et al., 2019 (Brazil)	In vitro	Dentin (Eroded) Human third molars 7 samples per group	Proanthocyanidin: 3%	CHX: 2%	Treatment approach – Incorporated into the phosphoric acid. Phosphoric acid contains or does not contain the active ingredients were applied for 30 s, rinsing for 15 s and drying in air, then the adhesive steps were carried out.	Microtensile bond strength, nanoleakage and in situ degree of conversion	Proanthocyanidin and CHX showed similar bond strength to eroded dentin, but proanthocyanidin performed better with Scotchbond and Tetric N-Bond adhesives.	(+)
Wang et al., 2018 (China)	In vitro	Enamel/dentin (Eroded) Human third Molars and premolars 10 samples per group	EGCG: 400 µg/m EGCG 400 µg/m + NaF 0.5 M	Placebo: Distilled water; NaF: 0.5 M	Preventive approach – Solution. Each tooth specimen was first immersed in 30 mL of artificial saliva for 6 h. After this period, the specimens were placed in 10 mL of the test solution for 4 min. Following the treatment, the specimens were rinsed with distilled water for 10 s. Prior to the erosive challenge, the specimens were again placed in artificial saliva for 6 h.	CLSM; Microhardness, SEM examination	EGCG and EGCG + NaF reduced microhardness loss, with EGCG being more effective than NaF, with no synergistic effect between them. Dentin showed greater loss of structure, while EGCG effectively reduced tubule exposure.	(+)
Boteon et al., 2017 (Brazil)	In vitro	Dentin (Sound) Bovine Teeth 8 samples per group	Proanthocyanidin: 0.05%,1%, and 5%	Negative control: Placebo	Preventive approach – Gels. The gels were applied to the dentin surface just once, prior to the initial erosive challenge. Each formulation shared the same base composition, differing only in proanthocyanidin (PA) concentration and application time, which was either 1–5 min.	Profilometric analysis	PA gels effectively reduced dentin wear, outperforming placebo gels, regardless of dose or application time. All PA gel formulations showed better performance in attenuating dentin erosion.	(+)
Yu et al., 2017 (China)	In vitro	Dentin (Sound) Human third Molars 16 samples per group	EGCG: 10 mg in 100 µL	Negative control: Placebo	Preventive approach – Toothpaste. Specimens were treated with a paste containing EGCG, which was applied twice to the occlusal surfaces of the dentin specimens for 30 s at low speed using a rotating cup. Following the various treatments, all specimens were stored in artificial saliva.	Dentin Permeability, CLSM, and SEM	EGCG-containing toothpaste effectively occluded dentinal tubules, reduced permeability, and enhanced resistance to acid and abrasion. It also sustained the release of EGCG, Ca, and P, showing strong inhibitory effects.	(+)
Kato et al., 2012 (Brazil)	In vitro	Dentin (Eroded) Bovine teeth 45 samples per group	EGCG: 400 µg/m	Placebo; NaF: 1.23%; CHX: 0.012%	Treatment approach – Gels. All gel formulations shared a similar composition, with the exception of the active compounds, and were applied in a thin layer using a microbrush for 1 min.	Collagen degradation by assaying hydroxyproline, dentin matrix loss.	Gels with EGCG, CHX significantly reduced hydroxyproline levels and dentin matrix loss, indicating their protective effect is linked to inhibiting degradation of the demineralized organic matrix.	(+)

*ATM *Atomic force microscopy, *CHX* chlorhexidine, *CLSM* Laser scanning confocal microscope, *DMSO* dimethyl sulfoxide, *DOM* Demineralized organic matrix, *EDL* Erosive dentin loss, *EGCG* Epigallocatechin-3-gallate, *ICTP* Carboxy-terminal telopeptide of type I collagen, *MMP* Matrix metalloproteinase, *QCT* Quercetin, *NaF* Sodium fluoride, *SEM* Scannin electron microscopy, *TEM* Transmission electron microscopy. *Grade of the treatment: (+) indicates a positive effect, and (=) indicates no difference compared to the control

The compiled data were synthesized into a narrative summary, emphasizing experimental protocols, intervention characteristics, key findings, and conclusions, thereby ensuring a clear, concise, and structured presentation of the results.

## Results

### Study selection

**Fig. 1:**
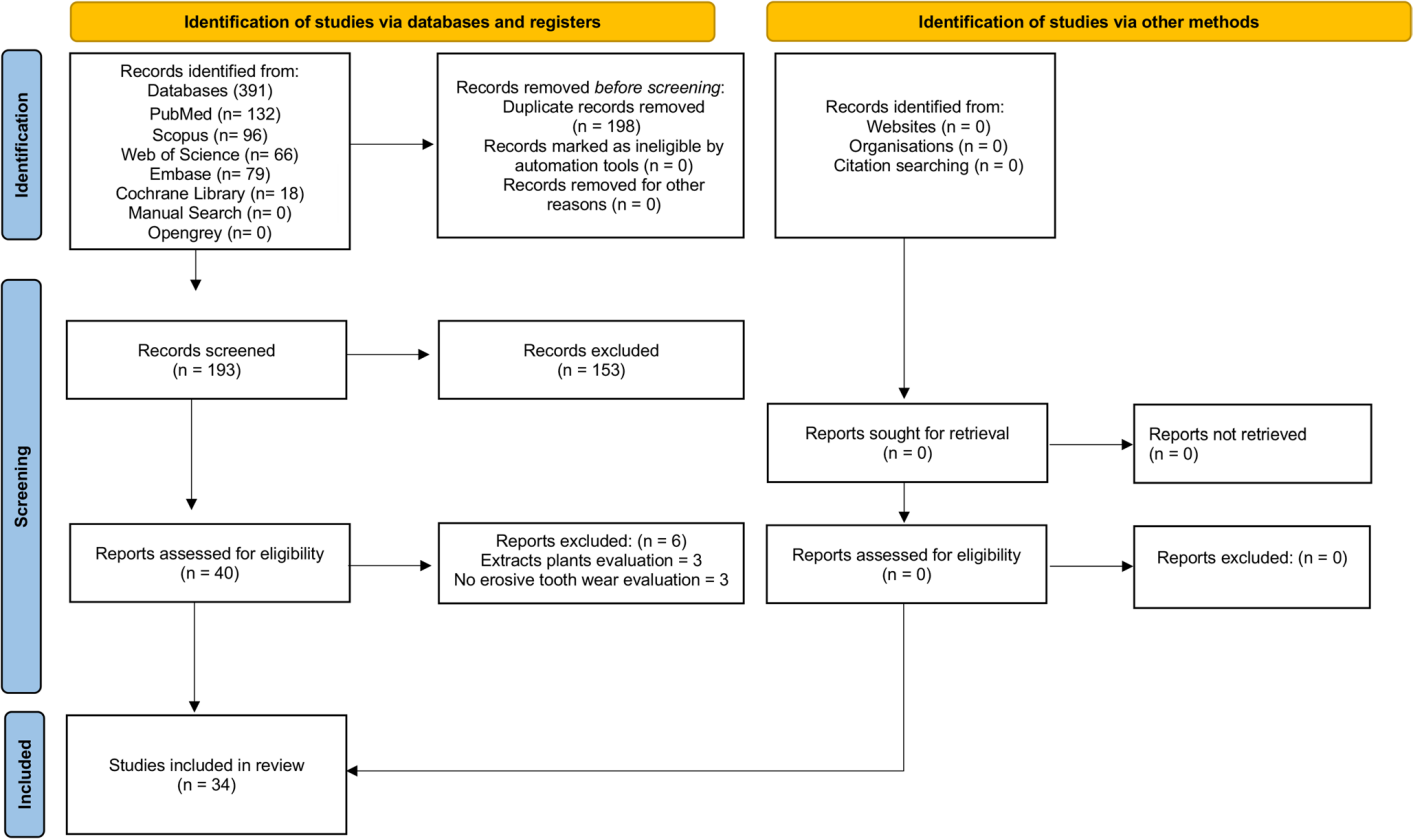
Flow Diagram of Study Selection According to PRISMA-ScR Guidelines

A total of 391 studies were initially identified: PubMed (*n* = 132), Scopus (*n* = 96), Web of Science (*n* = 66), Embase (*n* = 79), and Cochrane Library (*n* = 18). After removing 198 duplicates, 193 unique records remained for title and abstract screening. From these, 40 studies were selected for full-text reading. Following detailed evaluation, 6 studies were excluded for not meeting the inclusion criteria, and 34 studies were finally included in this scoping review [24–26, 36, 37, 42–70]. The details of the search strategy are illustrated in the flow diagram (Fig. 1). The kappa score for the articles included across all databases indicated an acceptable level of agreement between the reviewers (k = 0.96).

### Characteristics of the included studies

The studies included in this review were published between 2010 and 2024, with their characteristics summarized in Table 1. These eligible studies were conducted in various countries, including Brazil [24–26, 36, 44, 45, 48, 52–55, 58–60, 62–66, 69, 70], China [37, 42, 46, 47, 50, 56, 61, 67, 68], Pakistan [49], Turkey [51], United States of America [57], and Germany [64].

Concerning the study design, the included studies comprised two randomized clinical trials [44, 68], five in situ investigations [48, 53, 55, 59, 66], 20 in vitro experiments [24–26, 36, 37, 42, 45, 46, 49–51, 54, 56, 57, 60, 62, 63, 65, 67, 69], and seven studies conducted both in situ and in vitro approaches [43, 47, 52, 58, 61, 64, 70]. The majority of the included studies (79%; *n* = 27) focused on investigating the effects of flavonoids on dentin tissue [25]– [26, 37, 42, 44–46, 48, 51–53, 55–57, 61–65, 70]. In contrast, around 21% (*n* = 7) of the studies targeted enamel tissue specifically [37, 43, 49, 58, 64, 66].

Regarding the condition under investigation, all included studies evaluated erosive tooth wear. In addition, seven studies also considered abrasion as an associated factor [24, 42, 43, 47, 48, 53, 54], while one study specifically focused on abfraction lesions [44]. Flavonoids were predominantly investigated for their therapeutic potential, with 21 out of 34 included studies (65%) applying them as a treatment for existing erosive tooth wear lesions [24, 36, 42, 44–46, 48, 50–57, 59–61, 63, 68, 69]. In contrast, 13 studies (35%) were designed to investigate preventive approaches, aiming to protect sound dental tissues from erosive tooth wear [25, 26, 37, 43, 47, 49, 58, 62, 64–67, 70].

### Study designs, dental substrates, and experimental approaches

Regarding the study designs, two randomized clinical trials (included in this review reported divergent outcomes. In one study evaluating the effect of the flavonoid EGCG as a dentin pretreatment for abfraction lesions, a high restoration retention rate (93.3%) was observed; however, no significant effect on restoration survival was detected after 18 months [44]. In contrast, for eroded surfaces, the EGCG-treated group showed a faster and more pronounced reduction in ICTP expression compared to the control group, indicating decreased degradation of type I collagen matrix [68]. Overall, 10 of the 12 (83%) in situ investigations demonstrated positive effects on dental tissues [43, 47, 48, 55, 58, 61, 64, 66, 69, 70], particularly regarding outcomes such as reduced structural dental loss and inhibition of MMP activity. Among the 26 in vitro studies, 25 demonstrated protective and preventive effects [25, 26, 36, 37, 42, 45–47, 49–51, 54, 56–58, 60–65, 67, 69, 70], such as reduced dentin loss, improved mechanical properties, and enhanced control of erosion, as well as beneficial outcomes in procedures involving previously eroded dental tissues.

Regarding dentin as the substrate, 24 of the 27 studies evaluating dentin [24–26, 36, 42, 45–48, 50–57, 60–63, 65, 67, 69, 70] reported significant protective effects of flavonoids, including improvements in mechanical properties, enhanced dentin adhesion, reduction of structural loss due to demineralization, and promotion of remineralization. For enamel, all seven studies (*n* = 7) demonstrated positive outcomes following flavonoid application [37, 43, 49, 58, 64, 66], with effects primarily related to increased resistance to erosive tooth wear and modulation of protective salivary proteins, such as proline-rich proteins, calcium-binding proteins, and statherin [64, 66].

Concerning the use of flavonoids for the prevention and treatment of erosive tooth wear, 18 of the 21 treatment-focused studies (86%) reported a significant reduction in tissue loss [24, 36, 42, 45, 46, 48, 50, 51, 53–57, 60, 61, 63, 68, 69], including enhanced dentin adhesion when flavonoids were incorporated into conventional adhesive systems or applied as a pretreatment in restorative procedures [55, 60, 63]. Similarly, when evaluated for their preventive potential on sound dental tissues, all 13 studies addressing this condition reported protective effects against erosive challenges [25, 26, 37, 43, 47, 49, 58, 62, 64–67, 70].

In the context of the condition under investigation, of the 26 studies that analyzed the effects of flavonoids on dental erosion [24–26, 36, 37, 42–48, 50–55, 57, 59–63, 68, 69], 24 reported protective outcomes, demonstrating their ability to reduce tissue loss and to slow the progression of erosive challenges [24–26, 36, 37, 42–48, 50, 51, 53–55, 57, 60–63, 68, 69]. Moreover, seven studies also assessed abrasion as an additional factor [24, 42, 43, 47, 48, 53, 54], all of which highlighted the beneficial effects of flavonoids, particularly a reduction in dentin loss.

### Flavonoids administration, and measurements

Flavonoids were administered in different formulations, including treatment solutions [24, 25, 37, 42–44, 46, 47, 49, 50, 52, 54–57, 59–64], topical oral gels [26, 36, 45, 53, 58, 65, 66, 68–70], dentifrices [48, 67], and, in one study, as an additive in phosphoric acid for restorative procedures [63].

The duration of sample exposure to flavonoids varied among investigations. Short-term protocols involved exposure times ranging from 30 to 60 s [24–26, 43–46, 48, 51–56, 58, 62–64, 66, 67, 69, 70], and 2 to 5 min [37, 42, 47, 49, 50, 61]. Longer exposures reached up to 60 min [57, 58]. In addition, in one study, a tray-based application was used to maintain contact with the flavonoid overnight, simulating an 8 h exposure during sleep [68].

Among the main methods used to assess erosive tooth wear, profilometry was the most frequently employed technique across the included studies. In addition, microhardness tests were widely applied to investigate changes in the mechanical properties of dental substrates [25, 37, 42, 49, 52, 58–61]. To examine dentinal tubule permeability and surface morphological alterations, scanning electron microscopy (SEM) was extensively used [24–26, 37, 42, 46, 49–51, 54–56, 67, 70], along with confocal laser scanning microscopy (CLSM) in some studies [37, 42, 46, 67]. MMP activity was evaluated through zymography [25, 46, 47], while collagen degradation was assessed by quantifying the carboxy-terminal telopeptide of type I collagen (ICTP) [42, 45, 61, 68, 69]. Furthermore, adhesive performance was analyzed using bond strength tests to assess the effectiveness of restorative materials on eroded substrates [24, 36, 46, 47, 60, 62, 63].

### Flavonoids and control agents

Among the flavonoids studied, epigallocatechin-3-gallate (EGCG) was the most frequently investigated [24, 36, 37, 44, 46, 49, 51–54, 56, 57, 61, 62, 64, 66–70], followed by proanthocyanidin [26, 43, 45, 48, 55, 58–60, 63, 65], quercetin [25, 37, 42, 50, 61], theaflavin [46] and hesperidin [54].

The concentrations of flavonoids tested varied across studies. Most reported concentrations ranged from 10 to 100 µg/mL [25, 44, 49, 56, 61] 100 to 400 µg/mL [17, 37, 42, 47, 50, 53, 56, 57, 61, 64, 66, 69, 70], and up to 1000 µg/mL [55]. Some studies used flavonoid formulations with concentrations from 0.1 to 0.6% [24, 51, 52, 54, 55, 62], while others explored higher ranges: 1–3% [43, 46, 54, 64, 65, 68], 4–6.5% [55, 58, 60, 63, 65], and 8–10% [26, 45, 46, 48, 59]. All studies included negative controls, placebo (water) and/or untreated groups. Regarding positive controls, the main conventional agents identified were chlorhexidine (CHX) [26, 36, 46, 47, 50, 53, 59, 61–63, 66] and sodium fluoride (NaF) [25, 37, 42]. Both agents were also tested simultaneously in some studies [45, 48, 51, 69, 70]. Alternative positive controls included stannous fluoride [43] and potassium nitrate [49]. Concentrations used for CHX varied: 120 µg/mL [47, 50, 61], 0.012% [26, 48, 53, 59, 69, 70], 0.12% [36, 45, 66], and 1–2% [46, 51, 62, 63]. NaF concentrations included 1.23% [42, 45, 61, 69, 70], and 1000–1100 ppm [25, 57].

### Effects of isolated flavonoids on erosive tooth wear

#### Epigallocatechin-3-gallate (EGCG)

The potential of EGCG in the protection and repair of dental substrates affected by erosive tooth wear was explored in 20 of the included studies. Among these, 19 consistently reported favorable outcomes, especially in preventing dentin loss and enhancing substrate resilience [24, 36, 37, 46, 49, 51–54, 56, 57, 61, 62, 64, 66–70]. While one study, the neutral result was obtained when incorporated into the adhesive system [44].

When compared to CHX, EGCG presented equivalent or superior performance across several outcomes. Studies on dentin erosion and abrasion showed that EGCG, at concentrations of 400 µM and 0.6%, provided more effective protection than CHX (0.012%–2%), significantly minimizing tissue loss and surface damage [51, 53, 61]. Its remineralization potential was found to be similar to CHX [51, 54], while its ability to inhibit collagen degradation and stabilize the dentin matrix components appeared superior [68, 69]. Regarding adhesive performance, EGCG maintained microtensile bond strength over time in both sound and eroded dentin, whereas CHX was associated with reduced bonding durability [36, 46, 62]. In cases of tooth wear, such as abfraction lesions, EGCG demonstrated higher bond strength after three months of aging, reinforcing its potential to enhance long-term adhesion stability [24].

In comparative investigations with NaF (1100 ppm), EGCG (400 µg/mL- 0.6%) has demonstrated superior efficacy in performance dentin against erosive challenges and promoting remineralization [37, 51, 57, 70]. Some studies have reported a significant attenuation of tooth wear following EGCG application, highlighting its potential as a more effective alternative to conventional fluoride-based therapies [37, 51]. EGCG (400 µg/mL) also demonstrated a superior capacity to preserve dentin mechanical properties compared to NaF (0.05%) [52], likely due to its role in maintaining proteins essential for calcium and phosphate regulation [66]. In pH-cycling models, EGCG showed a pronounced protective effect on dentin erosive tooth wear, significantly reducing tissue loss compared to 1000 ppm NaF [57]. Furthermore, the use of EGCG-based gels (400 µM) inhibited dentin wear, exhibiting a protective capacity similar to that of CHX (0.012%) and markedly superior to both NaF (1.23%) treatments [70].

Among the various biomodifying agents studied, EGCG stood out for its promising performance, showing similar or even superior effectiveness to potassium nitrate in reducing erosive damage [49]. When its interaction with the salivary pellicle was evaluated, EGCG did not alter the surface charge but notably increased both the roughness and thickness of the pellicle, features that may strengthen its protective barrier. In contrast, while cationic astringents led to overcharging of the pellicle, and lysozyme significantly altered its electrostatic profile, these agents had limited impact on the pellicle’s surface architecture [64].

Regarding dentin permeability and tubular occlusion, pastes containing EGCG (100–400 µg/mL) exhibited a remarkable ability to occlude dentin tubules, reduce permeability, and enhance resistance to acid challenges [37, 56]. Furthermore, only one study explored the synergistic effect between EGCG (400 µg/mL) combined with NaF (0.5 M) [37]. Despite all groups showing some reduction in microhardness, the EGCG-treated samples exhibited the least reduction, demonstrating a possible protective synergy between EGCG and fluoride against erosive wear [37].

#### Proanthocyanidin

Proanthocyanidin (PA) was the second most investigated flavonoid, being present in 10 studies, all reporting protective effects against erosive and abrasive challenges [26, 43, 45, 48, 55, 58–60, 63, 65], Treatment with PA at concentrations ranging from 1% to 10% has been shown to significantly reduce erosive tooth wear across studies [26, 43, 45, 48, 59, 65]. Notably, PA demonstrated less enamel wear compared to CHX (0.012%–0.12%) [26, 45, 48] and NaF (1100 ppm; 1.23%) [45, 48]. Furthermore, the incorporation of 10% PA into mouthrinse formulations yielded a protective effect on enamel wear similar to CHX (0.012%) [59].

In the context of preventing erosive tooth wear, PA-based gels have also proven to be effective when applied topically [26, 58, 65]. The application of PA gels at concentrations of 0.05%, 1%, and 5% significantly reduced dentin wear, regardless of the concentration or application time (1–5 min) [65]. Under erosion-abrasion conditions, enamel treated with 2% PA showed wear levels similar to erosion-only groups, while offering protection comparable to SnCl₂/NaF/AmF formulations [43]. Although PA alone did not completely arrest lesion progression, its combination with TiF₄ varnish provided superior protection against erosive dentin lesions compared to chlorhexidine (0.012%) [26].

Regarding its anti-collagenolytic potential, 10% PA outperformed both CHX (0.12%) and NaF (1.23%) by significantly reducing collagen degradation, thereby contributing to dentin structural preservation and inhibition of enzymatic breakdown [45]. In restorative dentistry, PA has also demonstrated promising outcomes [36, 60, 63]. The incorporation of cross-linking primers containing 6.5% PA into adhesive protocols for eroded dentin significantly enhanced immediate bond strength and improved mechanical properties [60]. Similarly, the use of 3% PA led to a notable increase in the microtensile bond strength of eroded dentin when compared to 2% CHX, especially when applied in combination with universal adhesive systems [63].

#### Quercetin

Quercetin was exclusively evaluated on dentin tissue and investigated in both preventive and therapeutic contexts. In all studies, it consistently demonstrated protective and reparative effects against dentin erosion and wear [25, 37, 42, 50, 61].

In preventive applications, quercetin at 0.03% has shown remarkable potential in preventive strategies, effectively cross-linking dentin collagen and partially inhibiting MMP-2 and MMP-9 activity, achieving outcomes similar to 1100 ppm NaF [25]. Among the tested agents, quercetin-treated dentin exhibited the highest surface hardness, significantly outperforming NaF alone. Interestingly, quercetin combined with NaF synergistically enhanced dentin protection by strengthening mechanical resilience and fully inhibiting enzymatic collagen degradation [25]. Compared to CHX (120 µg/mL), quercetin (75–300 µg/mL) exhibited superior efficacy in preventing dentin erosion, with greater reduction in tissue loss and enhanced mechanical properties of dentin [47]. This protective effect was attributed to a dose-dependent increase in collagen cross-linking within the tested concentration range [47]. Additionally, quercetin contributed to improved microtensile bond strength to dentin, suggesting an additional benefit in reinforcing adhesive performance alongside its anti-erosive properties [47].

In cases of pre-established erosive tooth wear, quercetin (300 µg/mL) demonstrated greater effectiveness in controlling lesion progression compared to sodium fluoride at 1.23% [25, 50, 61], and outperformed CHX at 120 µg/mL [61] or showed similar effects [50]. When compared to another well-studied flavonoid, EGCG, quercetin also proved to be more effective [61]. Interestingly, quercetin was effective under both erosive challenges alone [50, 61] and combined erosive-abrasive conditions [42].

Moreover, dentin treated with 300 µg/mL quercetin exhibited superior tubule occlusion compared to NaF (1.23%) and CHX (120 µg/mL), with fewer exposed tubules observed under SEM analysis [42, 50]. Additionally, quercetin enhanced dentin mechanical properties [42, 50, 61], supporting its role in preserving structural integrity. In terms of organic matrix preservation, it significantly reduced collagen degradation [42, 61], with collagen cross-linking increasing proportionally to concentration (75–300 µg/mL) [61]. At 300 µg/mL, quercetin markedly outperformed lower doses, reducing demineralized organic matrix more effectively than both CHX and NaF controls. While MMP inhibition did not differ significantly among treatments, quercetin provided greater protection against dentin erosion without compromising the aesthetic appearance of the substrate [61].

#### Other flavonoids

Other flavonoids, such as theaflavin [46] and hesperidin [54], have been explored in isolated studies and showed promising, albeit varied, effects. Theaflavin was evaluated at concentrations of 1%, 2%, 4%, and 8%, demonstrating a clear dose-dependent protective effect against the progression of erosive tooth wear [46]. Dentin surfaces treated with theaflavin exhibited significantly reduced wear compared to the negative control, with more pronounced effects observed even at lower concentrations. In addition to reducing wear, theaflavins inhibited matrix metalloproteinase activity, promoted collagen cross-linking, and enhanced the hydrophilic properties of dentin collagen. Consequently, the collagen cross-linking potential of theaflavin has been shown to increase microtensile bonding of the dentin organic matrix [46].

These effects surpassed those of 1% CHX and were comparable to those observed with 1% EGCG [46].

Hesperidin, tested at 0.1%, 0.5%, and 1%, also demonstrated protective effects, though to a lesser extent than EGCG. While all concentrations contributed to a reduction in dentin wear, the protective potential was modest compared to 0.46% EGCG [54]. Indeed, higher concentrations of hesperidin promoted dentinal tubule occlusion, suggesting a concentration-dependent effect. At 1%, hesperidin preserved the demineralized organic matrix layer and effectively protected against citric acid-induced erosion. Additionally, hesperidin reduced both erosive and abrasive wear across all tested concentrations, although its efficacy remained below that of EGCG under both erosive and erosive–abrasive conditions [54].

## Discussion

**Fig. 2:**
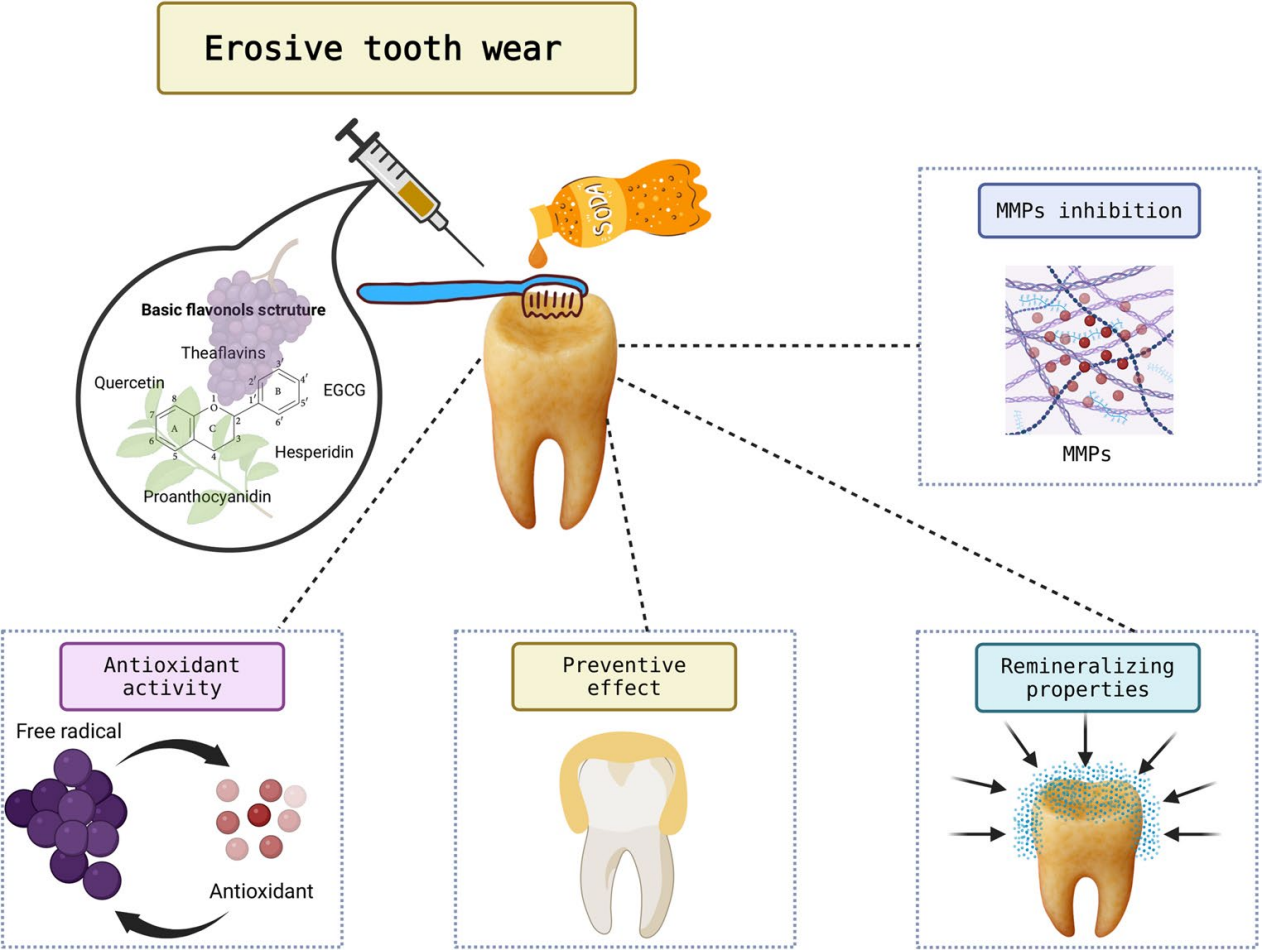
Schematic illustration of the effects of flavonoids on erosive tooth wear

This scoping review aimed to examine the impact of flavonoids on erosive tooth wear, with a focus on their potential preventive and therapeutic effects on dental substrates. Based on the analysis of 34 studies, flavonoids have been identified as promising phytotherapeutic agents, acting through multiple mechanisms to protect against erosive tooth wear. The review indicates that these compounds can enhance the mechanical properties of mineralized dental tissues, contributing to the reduction of structure loss due to demineralization, as well as supporting remineralization processes on previously affected surfaces. In addition, flavonoids have also demonstrated the ability to inhibit enzymatic degradation of the dentinal organic matrix, thereby attenuating the deleterious effects of protease activation during erosive attacks. Another relevant aspect is their influence on the salivary pellicle, inducing morphological and structural changes that may increase its resistance to acidic challenges. These effects, observed across various experimental models, reinforce the potential of flavonoids as adjunctive/novel agents in the prevention and management of erosive tooth wear (Fig. 2).

The favorable outcomes observed in this review are directly linked to the biological properties of flavonoids, bioactive compounds classified as secondary plant metabolites, characterized by their polyphenolic structure and low molecular weight [71]. Flavonoids exhibit multiple mechanisms of action that contribute to the protection and repair of dentin in the face of erosive and abrasive challenges. One of the main reported effects is their ability to promote collagen cross-linking, thereby reinforcing the dentin organic matrix and enhancing its mechanical strength and stability against enzymatic degradation [25, 42, 69]. In addition, flavonoids such as EGCG and quercetin show potent inhibitory effects on MMPs, enzymes responsible for collagen breakdown, which slows the loss of the organic matrix during dentin erosion processes [25, 47, 61]. This preservation of the organic matrix has been associated with the maintenance of dentin microhardness and structural integrity, facilitating remineralization and protection against tissue loss. Another relevant mechanism is the ability of flavonoids to modify the dentin surface by promoting the occlusion of dentinal tubules and reducing permeability, which limits acid penetration and erosion progression [50, 56].

Furthermore, the interaction between flavonoids and the salivary pellicle contributes to an increase in the thickness and roughness of the protective film, enhancing the physical barrier against erosive agents [64]. These multiple mechanisms highlight the potential of flavonoids as biomodifying agents capable of acting at different stages of the etiology of erosive tooth wear. Additionally, it is important to note that although most studies focus on dentin, evidence also points to benefits of flavonoids on dental enamel [37,43,49,58,64,]. In this tissue, flavonoids act by inhibiting demineralization and promoting remineralization, leading to the formation of a protective layer that reduces mineral solubility under acidic conditions [49, 58, 72]. Their chelating properties facilitate the retention of calcium ions on the enamel surface, while their antioxidant activity neutralizes free radicals that could otherwise intensify erosive damage.

The data obtained reinforces the synergistic influence of erosive and abrasive challenges in intensifying tooth wear, with significantly greater losses being observed compared to protocols with erosion alone. This finding corroborates the consolidated literature, which points out that acid challenge promotes softening of the dentin surface, partially removing the hydroxyapatite crystals and exposing the organic matrix, making it more vulnerable to the subsequent mechanical action of brushing [73–75]. Brushing previously demineralized dentin increases wear, mainly by removing the thin residual collagen layer which, under ideal conditions, could act as a temporary protective barrier. In this context, the superior efficacy of flavonoids compared to conventional treatments with NaF and CHX, as well as the placebo group, is also noteworthy, and the significant differences can be attributed to the ability of flavonoids to preserve the organic matrix of dentin. The collagen stabilization effect promoted by compounds such as EGCG, hesperidin, quercetin, and proanthocyanidins prevents the direct exposure of mineralized dentin to mechanical challenges, which may explain the increased resistance to wear from brushing, as reported in the literature [76]. Thus, flavonoids not only attenuate the erosive impact, but also offer additional protection against subsequent abrasive insults - an important differential compared to conventional fluoride-based treatments, which act predominantly on the mineral component, but have a less pronounced effect on the conservation of the organic matrix.

In cases where erosive tooth wear is already established and restorative treatment is indicated, composite resins are generally the material of choice for restoring lost dental structure [77, 78]. However, the durability of the resin–dentin bond may be compromised by the degradation of the hybrid layer, especially due to the action of MMPs [79], highlighting the need for complementary strategies to enhance adhesive interface stability [80, 81]. In this scoping review, various flavonoids were evaluated for their therapeutic potential on eroded dentin substrates, with emphasis on collagen matrix protection and adhesive bond stabilization [24, 36, 55, 60, 63, 82], EGCG was among the most studied compounds, demonstrating superior performance compared to CHX in preserving adhesive strength in eroded dentin [36, 62], an effect attributed to its antioxidant properties and MMP-inhibitory action [24]. Proanthocyanidins also showed relevant performance by inhibiting MMPs and promoting dentin collagen cross-linking, resulting in improved adhesive interface stability [45, 55, 60, 63]. Their mechanism of action appears to involve interaction with the active or allosteric sites of proteolytic enzymes [45]. Quercetin, in turn, exhibited a dose-dependent effect in reducing dentin loss and improving mechanical strength, favoring resin–dentin bond stability without compromising aesthetics [47]. These effects are attributed to its ability to cross-link the organic matrix and interact with dentin structural proteins. Lastly, theaflavins stood out for their potent antiproteolytic activity [46], associated with their structure rich in phenolic and galloyl groups, similar to that of EGCG [46, 56], contributing to the reduction in the progression of dentin erosion. Thus, the flavonoids evaluated in this review also demonstrated bioactive properties that significantly contribute to the protection and stabilization of eroded substrates, positioning them as promising adjuncts in restorative therapies for erosive tooth wear.

The efficacy of flavonoids in controlling erosive tooth wear exhibits a distinctly dose-dependent pattern, where higher concentrations tend to enhance the protective effects of these compounds [46, 47, 52, 54, 61, 64, 65, 68]. Proanthocyanidin, for instance, as evidenced in the eligible studies, has been recognized as effective within the 1% to 10% range, with superior performance observed at higher concentrations [65] suggesting a direct correlation between dose and efficacy when compared to conventional agents such as CHX and NaF [45, 48]. Similarly, quercetin shows promising potential to strengthen the dentin matrix by promoting collagen cross-linking and inhibiting MMPs, particularly at intermediate concentration ranges, which may account for its more pronounced protective action under erosive conditions [25, 47, 61]. In addition, theaflavin and hesperidin also demonstrated dose-dependent protective effects against erosive tooth wear [46, 54]. In the case of EGCG, although its functional properties are evident even at lower concentrations [52, 64, 70], the literature suggests that its efficacy can also be enhanced at higher concentrations, especially when applied to dentin tissue, thus contributing to improved structural integrity in response to erosive challenges [51, 52, 68]. However, it is important to note that formulations with higher concentrations must be carefully evaluated due to the potential risk of adverse effects and the need to ensure biocompatibility, factors that remain insufficiently addressed in the current literature. Therefore, precise concentration adjustment emerges as a critical factor for maximizing the therapeutic benefits of flavonoids, representing a key element in the development of safe, effective, and evidence-based clinical formulations.

The control of erosive tooth wear is challenging in mineralized dental tissues; however, this complexity is greater in dentin due to its lower mineral content and high collagen composition [17]. The process involves not only the dissolution of inorganic components but also the exposure of the demineralized organic matrix [82]. Although this matrix can act as a physical barrier, limiting ion diffusion and mitigating acid damage [83], its integrity is compromised by the activity of MMPs, which is enhanced in acidic environments, contributing to proteolytic degradation of dentin and progression of erosive tooth wear [54]. Therapeutic strategies incorporating bioactive agents with remineralizing properties and MMP inhibitory capacity have shown promising results in preserving dentin integrity against erosive challenges [84]. In the studies included in this scoping review, commonly used compounds such as chlorhexidine and sodium fluoride were frequently employed as positive controls, highlighting their recognized efficacy in protecting the dentin organic matrix. However, CHX, despite its effectiveness, has clinical limitations and limited remineralizing activity, along with challenges related to treatment adherence and inability for continuous use due to adverse effects such as taste alteration, oral burning, xerostomia, and dental staining, typically occurring after three weeks of continuous use [85]. Fluoride, on the other hand, primarily acts superficially by forming a calcium fluoride layer on the dental surface [86] nevertheless, this protection is limited against aggressive and recurrent acid exposures and exhibits low penetration into dentin tissues. Given dentin’s higher organic content, fluoride exerts only a restricted effect on the already exposed organic matrix, as well as a reduced antiproteolytic effect [87, 88]. Clinically, this limitation underscores the need for adjunctive strategies that target both the mineral and organic components of dentin, as fluoride alone may be insufficient to halt lesion progression or preserve dentin integrity under severe erosive conditions. Moreover, despite its widespread use and proven efficacy, excessive ingestion of fluoridated products can lead to adverse effects, including dental fluorosis, and, in rare instances, acute intoxication, particularly in pediatric populations due to difficulties in expectoration and insufficient supervision [89, 90]. In this context, flavonoids emerge as promising alternatives due to their lower incidence of adverse effects [91], potent antiproteolytic activity, and capacity to act directly on the demineralized organic matrix [26, 45, 47, 61]. Several studies have demonstrated that these compounds perform equivalently or even superiorly to conventional agents in protecting the collagen matrix against enzymatic degradation [45, 47, 69, 70], reinforcing their potential as an effective and safe clinical alternative for controlling erosive tooth wear. Moreover, as evidenced in the eligible studies, the combination of NaF with different flavonoids, such as EGCG [37] quercetin [25], and proanthocyanidin [48] demonstrated enhanced formulation effects, highlighting the synergistic action between these bioactive agents. These findings reinforce the feasibility of adjuvant therapeutic strategies with greater potential for managing erosive tooth wear.

Despite the promising protective and reparative effects of flavonoids on dental tissues, it is essential to emphasize that the management of erosive tooth wear should follow a comprehensive clinical approach, ranging from early diagnosis to individualized strategies for prevention and progression control [92]. Identifying the initial signs of erosion requires the clinician’s careful attention, as its manifestations are often subtle and asymptomatic. Moreover, a thorough assessment of each patient’s specific risk factors is crucial, including dietary habits, frequency and mode of acidic substance intake, the presence of gastroesophageal reflux or eating disorders, as well as the quantity and composition of salivary flow [2, 5]. In this context, flavonoids can be incorporated as adjuncts to preventive and therapeutic measures, as they contribute to dentin protection by inhibiting MMPs, cross-linking collagen, and stabilizing the organic matrix [25, 42]. They also promote beneficial effects on enamel, such as calcium ion retention and potential antioxidant activity against demineralization [64, 71]. However, it is important to emphasize that their use must be integrated into appropriate clinical protocols, including patient education on reducing acid exposure, the rational use of fluorides, salivary stimulation, and the adoption of protective habits. The combination of behavioral interventions, topical therapies, and biomodifying agents, such as flavonoids, represents a promising strategy for the effective control of erosive tooth wear and the long-term preservation of dental hard tissues.

Although this scoping review provides a comprehensive overview of the use of flavonoids in the context of erosive tooth wear, certain limitations must be acknowledged when interpreting the findings. Most of the studies analyzed were conducted in vitro, using models that simulate tooth wear in laboratory conditions, which may not fully reflect the complexity of the oral environment. Another limitation concerns the marked methodological heterogeneity among the included studies. While all reported beneficial effects associated with flavonoid interventions, there was substantial variability in experimental models (in vitro, in situ, and clinical studies), intervention characteristics such as concentrations, modes of administration, exposure times, and outcome assessment methods, as well as in the substrates employed. In particular, thirteen studies used bovine teeth in their experimental protocols. Although bovine teeth are a well-established substitute in dental research due to their availability, standardization, and structural similarities to human teeth, they may not perfectly replicate human dental tissue responses, as bovine enamel has been reported to be more susceptible to erosive challenges [93]. Additionally, heterogeneity in erosion-inducing protocols—such as citric acid cycles or soft drinks—may have introduced further variability in outcomes, limiting direct comparability between studies [94]. Despite the data were synthesized separately for each flavonoid, this methodological diversity limits direct comparability between studies and hinders the ability to draw robust and consistent conclusions regarding the efficacy of each compound. Moreover, most studies were conducted in laboratory settings, and there remains a lack of well-designed clinical trials, which restricts the generalizability of the results to clinical practice.

Nevertheless, the findings compiled in this review are promising and highlight the potential of flavonoids as innovative adjuncts in the management of erosive tooth wear. These results support further investigation into the development of new formulations and the incorporation of these compounds alongside conventional preventive strategies, particularly in the absence of a universally accepted gold standard protocol for this condition. Future research should prioritize the standardization of flavonoid-based formulations (e.g., gels, toothpaste, varnishes, topical solutions, or restorative materials) and the definition of optimal usage parameters, including concentration, application frequency, and duration. The implementation of randomized controlled trials with representative samples, clearly defined clinical and radiographic outcomes, and long-term follow-up is essential to confirm the efficacy and safety of these agents across different populations. Comparative studies examining flavonoids alone or in combination with established agents, such as fluorides or calcium-based bioactive compounds, may clarify potential synergistic effects. Furthermore, investigations into the mechanisms of action, formulation stability, and feasibility of large-scale implementation are warranted to support the integration of evidence-based approaches into the clinical management of erosive tooth wear.

## Conclusion

Based on the aforementioned considerations, it can be concluded that flavonoids offer significant protective effects against erosive tooth wear and play an effective role in managing this condition, particularly by enhancing restorative procedures through dentin biomodification. Although most current evidence derives from studies using pre-eroded substrates, flavonoids also show preventive potential by reducing enamel and dentin softening at the onset of erosive challenges. Overall, these findings position flavonoids as a promising strategy for both the prevention and management of erosive tooth wear.

## Supplementary Information

Below is the link to the electronic supplementary material.

Supplementary Material 1

## Data Availability

No datasets were generated or analysed during the current study.
