# Supplementary material for: Therapeutic potential of flavonoids in erosive tooth wear management: a scoping review
Source: Clin Oral Investig. 2025 Oct 27;29(11):535. doi: 10.1007/s00784-025-06623-8 (PMC12559054; doi:10.1007/s00784-025-06623-8)
Supplement: Supplementary file 1 — Supplementary Material 1 [file 784_2025_6623_MOESM1_ESM.docx]

# Supplementary Files

**File 1. Search strategies used for the electronic databases**

| Search Strategy |
| --- |
| PUBMED/MEDLINE: 132  ((Tooth Wear) OR (Tooth Wears) OR (Wears, Tooth) OR (Wear, Tooth) OR (Dental Wear) OR (Dental Wears) OR (Wear, Dental) OR (Wears, Dental) OR (Enamel wear) OR (Dentin Wear) OR (Erosion, Tooth) OR (Erosive Tooth Wear) OR (Erosive Dentin Wear) OR (Tooth Erosion) OR (Tooth Erosions) OR (Dental Erosion) OR (Dental Erosions) OR (Erosion, Dental) OR (Dental Enamel Erosion) OR (Dental Enamel Erosions) OR (Enamel Erosion, Dental) OR (Erosion, Dental Enamel) OR (Eroded Enamel) OR (Dentin Erosion) OR (Eroded Dentin) OR (Abrasion, Tooth) OR (Abrasion, Dental) OR (Dental Abrasion) OR (Anti-erosive) OR (Protection Erosion)) AND ((Flavonoid) OR (Flavonoids) OR (Bioflavonoids) OR (Bioflavonoid) OR (Flavon) OR (Isoflavones) OR (Flavanones) OR ( Flavanols) OR (Flavanonols) OR (2-Phenyl-Chromene) OR (2 Phenyl Chromene) OR (2-Phenyl-Benzopyran) OR (2 Phenyl Benzopyran) OR (2-Phenyl-Benzopyrans) OR (2 Phenyl Benzopyrans) OR (2-Phenyl-Chromenes) OR (2 Phenyl Chromenes) OR (Proanthocyanidin) OR (Polyhydroxyflavan-3-ol) OR (Naringenin) OR (4',5,7-trihydroxyflavanone) OR (myricetin) OR (3,3',4',5,5',7-hexahydroxyflavone) OR (Hesperitin) OR (Hesperidin) OR (Taxifolin) OR (Dihydroquercetin) OR (Quercetin) OR (Quercetine) OR (Dikvertin) OR (3,3',4',5,7-Pentahydroxyflavone) OR (Isoquercitrin) OR (Quercetin-3-O-glucoside) OR (isoquercetin) OR (isoquercitin) OR (isotrifoliin) OR (epigallocatechin-3-gallate) OR (epigallo-catechin gallate) OR (epigallocatechin-3-O-gallate) OR (EGCG) OR (Ampelopsin) OR (Rutin) OR (Rutoside) OR (Morina) OR (Kaempferol) OR (Kaempferols) OR (Myricetin) OR (Epicatechin) OR (Catechin) OR (Isorhamnetin) OR (Fisetin) OR (3,3',4',7-Tetrahydroxy-flavone) OR (Chrysin) OR (chrysene) OR (5,7-dihydroxyflavone) OR (Pinocembrin) OR (eriodictyol) OR (Abyssinones) OR (Theaflavin) OR (Genistein) OR (Genestein) OR (Daidzein) OR (Theaflavin) OR (Macluraxanthone) OR (Scopoletin) OR (Luteolin) OR (3',4',5,7-Tetrahydroxyflavone) OR (Luteoline) OR (Apigenin))  (<http://www.ncbi.nlm.nih.gov/pubmed>)  SCOPUS: 96  (( TITLE-ABS-KEY (( tooth AND wear) OR (tooth AND wears) OR ( wears, AND tooth ) OR (wear, AND tooth) OR (dental AND wear) OR (dental AND wears) OR (wear, AND dental) OR (wears, AND dental) OR (enamel AND wear) OR (dentin AND wear) OR (erosion, AND tooth) OR (erosive AND tooth AND wear) OR (erosive AND dentin AND wear ) OR (tooth AND erosion) OR (tooth AND erosions) OR (dental AND erosion) OR (dental AND erosions) OR (erosion, AND dental) OR (dental AND enamel AND erosion) OR (dental AND enamel AND erosions) OR (enamel AND erosion, AND dental) OR (erosion, AND dental AND enamel) OR (eroded AND enamel) OR (dentin AND erosion) OR (eroded AND dentin) OR (abrasion, AND tooth) OR (abrasion, AND dental) OR (dental AND abrasion) OR (anti-erosive ) OR (protection AND erosion)) AND TITLE-ABS-KEY (( flavonoid) OR (flavonoids) OR ( bioflavonoids ) OR (bioflavonoid ) OR ( flavon ) OR ( isoflavones ) OR (flavanones) OR (flavanols) OR (flavanonols) OR (2-phenyl-chromene) OR (2 phenyl AND chromene ) OR (2-phenyl-benzopyran) OR (2 phenyl AND benzopyran ) OR ( 2-phenyl-benzopyrans) OR (2 phenyl AND benzopyrans) OR (2-phenyl-chromenes) OR (2 phenyl AND chromenes) OR (proanthocyanidin ) OR ( polyhydroxyflavan-3-ol) OR (naringenin) OR (4',5,7-trihydroxyflavanone) OR (myricetin) OR (3,3',4',5,5',7-hexahydroxyflavone) OR (hesperitin) OR (hesperidin) OR (taxifolin) OR (dihydroquercetin) OR (quercetin) OR (quercetine) OR (dikvertin) OR (3,3',4',5,7-pentahydroxyflavone) OR (isoquercitrin) OR (quercetin-3-o-glucoside) OR (isoquercetin) OR (isoquercitin) OR (isotrifoliin) OR (epigallocatechin-3-gallate ) OR (epigallo-catechin AND gallate) OR (epigallocatechin-3-o-gallate) OR (EGCG) OR (ampelopsin) OR (rutin) OR (rutoside) OR (morina) OR (kaempferol) OR (kaempferols) OR (myricetin) OR (epicatechin) OR (catechin ) OR (isorhamnetin) OR (fisetin) OR (3,3',4',7-tetrahydroxy-flavone) OR ( chrysin) OR (chrysene) OR (5,7-dihydroxyflavone) OR (pinocembrin) OR (eriodictyol) OR (abyssinones) OR (theaflavin) OR (genistein) OR (genestein) OR (daidzein) OR ( theaflavin) OR ( macluraxanthone) OR (scopoletin) OR (luteolin) OR (3',4',5,7-tetrahydroxyflavone) OR (luteoline) OR (apigenin)))  ([http://www.scopus.com](http://www.scopus.com/))  EMBASE: 79  ('tooth wear'/exp OR 'tooth wear' OR (('tooth'/exp OR tooth) AND ('wear'/exp OR wear)) OR 'tooth wears' OR (('tooth'/exp OR tooth) AND wears) OR 'wears, tooth' OR (wears, AND ('tooth'/exp OR tooth)) OR 'wear, tooth' OR (wear, AND ('tooth'/exp OR tooth)) OR 'dental wear' OR (('dental'/exp OR dental) AND ('wear'/exp OR wear)) OR 'dental wears' OR (('dental'/exp OR dental) AND wears) OR 'wear, dental' OR (wear, AND ('dental'/exp OR dental)) OR 'wears, dental' OR (wears, AND ('dental'/exp OR dental)) OR 'enamel wear' OR (('enamel'/exp OR enamel) AND ('wear'/exp OR wear)) OR 'dentin wear' OR (('dentin'/exp OR dentin) AND ('wear'/exp OR wear)) OR 'erosion, tooth'/exp OR 'erosion, tooth' OR (('erosion,'/exp OR erosion,) AND ('tooth'/exp OR tooth)) OR 'erosive tooth wear'/exp OR 'erosive tooth wear' OR (erosive AND ('tooth'/exp OR tooth) AND ('wear'/exp OR wear)) OR 'erosive dentin wear' OR (erosive AND ('dentin'/exp OR dentin) AND ('wear'/exp OR wear)) OR 'tooth erosion'/exp OR 'tooth erosion' OR (('tooth'/exp OR tooth) AND ('erosion'/exp OR erosion)) OR 'tooth erosions' OR (('tooth'/exp OR tooth) AND erosions) OR 'dental erosion'/exp OR 'dental erosion' OR (('dental'/exp OR dental) AND ('erosion'/exp OR erosion)) OR 'dental erosions' OR (('dental'/exp OR dental) AND erosions) OR 'erosion, dental' OR (('erosion,'/exp OR erosion,) AND ('dental'/exp OR dental)) OR 'dental enamel erosion' OR (('dental'/exp OR dental) AND ('enamel'/exp OR enamel) AND ('erosion'/exp OR erosion)) OR 'dental enamel erosions' OR (('dental'/exp OR dental) AND ('enamel'/exp OR enamel) AND erosions) OR 'enamel erosion, dental' OR (('enamel'/exp OR enamel) AND ('erosion,'/exp OR erosion,) AND ('dental'/exp OR dental)) OR 'erosion, dental enamel' OR (('erosion,'/exp OR erosion,) AND ('dental'/exp OR dental) AND ('enamel'/exp OR enamel)) OR 'eroded enamel'/exp OR 'eroded enamel' OR (eroded AND ('enamel'/exp OR enamel)) OR 'dentin erosion' OR (('dentin'/exp OR dentin) AND ('erosion'/exp OR erosion)) OR 'eroded dentin' OR (eroded AND ('dentin'/exp OR dentin)) OR 'abrasion, tooth' OR (('abrasion,'/exp OR abrasion,) AND ('tooth'/exp OR tooth)) OR 'abrasion, dental' OR (('abrasion,'/exp OR abrasion,) AND ('dental'/exp OR dental)) OR 'dental abrasion'/exp OR 'dental abrasion' OR (('dental'/exp OR dental) AND ('abrasion'/exp OR abrasion)) OR 'anti erosive' OR 'protection erosion' OR (('protection'/exp OR protection) AND ('erosion'/exp OR erosion))) AND (flavonoid:ti,ab,kw OR flavonoids:ti,ab,kw OR bioflavonoids:ti,ab,kw OR bioflavonoid:ti,ab,kw OR flavon:ti,ab,kw OR isoflavones:ti,ab,kw OR flavanones:ti,ab,kw OR flavanols:ti,ab,kw OR flavanonols:ti,ab,kw OR '2 phenyl chromene':ti,ab,kw OR '2 phenyl benzopyran':ti,ab,kw OR '2 phenyl benzopyrans':ti,ab,kw OR '2 phenyl chromenes':ti,ab,kw OR proanthocyanidin:ti,ab,kw OR 'polyhydroxyflavan 3 ol':ti,ab,kw OR naringenin:ti,ab,kw OR '4,5,7 trihydroxyflavanone':ti,ab,kw OR '3,3,4,5,5,7 hexahydroxyflavone':ti,ab,kw OR hesperitin:ti,ab,kw OR hesperidin:ti,ab,kw OR taxifolin:ti,ab,kw OR dihydroquercetin:ti,ab,kw OR quercetin:ti,ab,kw OR quercetine:ti,ab,kw OR dikvertin:ti,ab,kw OR '3,3,4,5,7 pentahydroxyflavone':ti,ab,kw OR isoquercitrin:ti,ab,kw OR 'quercetin 3 o glucoside':ti,ab,kw OR isoquercetin:ti,ab,kw OR isoquercitin:ti,ab,kw OR isotrifoliin:ti,ab,kw OR 'epigallocatechin 3 gallate':ti,ab,kw OR 'epigallo-catechin gallate':ti,ab,kw OR 'epigallocatechin 3 o gallate':ti,ab,kw OR egcg:ti,ab,kw OR ampelopsin:ti,ab,kw OR rutin:ti,ab,kw OR rutoside:ti,ab,kw OR morina:ti,ab,kw OR kaempferol:ti,ab,kw OR kaempferols:ti,ab,kw OR myricetin:ti,ab,kw OR epicatechin:ti,ab,kw OR catechin:ti,ab,kw OR isorhamnetin:ti,ab,kw OR fisetin:ti,ab,kw OR '3,3,4,7 tetrahydroxy flavone':ti,ab,kw OR chrysin:ti,ab,kw OR chrysene:ti,ab,kw OR '5,7 dihydroxyflavone':ti,ab,kw OR pinocembrin:ti,ab,kw OR eriodictyol:ti,ab,kw OR abyssinones:ti,ab,kw OR genistein:ti,ab,kw OR genestein:ti,ab,kw OR daidzein:ti,ab,kw OR theaflavin:ti,ab,kw OR macluraxanthone:ti,ab,kw OR scopoletin:ti,ab,kw OR luteolin:ti,ab,kw OR '3,4,5,7 tetrahydroxyflavone':ti,ab,kw OR luteoline:ti,ab,kw OR apigenin:ti,ab,kw)  ([https://www.embase.com](https://www.embase.com/))  WEB OF SCIENCE: 66  (Tooth Wear) OR (Tooth Wears) OR (Wears, Tooth) OR (Wear, Tooth) OR (Dental Wear) OR (Dental Wears) OR (Wear, Dental) OR (Wears, Dental) OR (Enamel wear) OR (Dentin Wear) OR (Erosion, Tooth) OR (Erosive Tooth Wear) OR (Erosive Dentin Wear) OR (Tooth Erosion) OR (Tooth Erosions) OR (Dental Erosion) OR (Dental Erosions) OR (Erosion, Dental) OR (Dental Enamel Erosion) OR (Dental Enamel Erosions) OR (Enamel Erosion, Dental) OR (Erosion, Dental Enamel) OR (Eroded Enamel) OR (Dentin Erosion) OR (Eroded Dentin) OR (Abrasion, Tooth) OR (Abrasion, Dental) OR (Dental Abrasion) OR (Anti-erosive) OR (Protection Erosion) (Topic) and (Flavonoid) OR (Flavonoids) OR (Bioflavonoids) OR (Bioflavonoid) OR (Flavon) OR (Isoflavones) OR (Flavanones) OR ( Flavanols) OR (Flavanonols) OR (2-Phenyl-Chromene) OR (2 Phenyl Chromene) OR (2-Phenyl-Benzopyran) OR (2 Phenyl Benzopyran) OR (2-Phenyl-Benzopyrans) OR (2 Phenyl Benzopyrans) OR (2-Phenyl-Chromenes) OR (2 Phenyl Chromenes) OR (Proanthocyanidin) OR (Polyhydroxyflavan-3-ol) OR (Naringenin) OR (4',5,7-trihydroxyflavanone) OR (myricetin) OR (3,3',4',5,5',7-hexahydroxyflavone) OR (Hesperitin) OR (Hesperidin) OR (Taxifolin) OR (Dihydroquercetin) OR (Quercetin) OR (Quercetine) OR (divertin) OR (3,3',4',5,7-Pentahydroxyflavone) OR (Isoquercitrin) OR (Quercetin-3-O-glucoside) OR (isoquercetin) OR (isoquercitrin) OR (isotrifoliol) OR (epigallocatechin-3-gallate) OR (epigallo-catechin gallate) OR (epigallocatechin-3-O-gallate) OR (EGCG) OR (Ampelopsin) OR (Rutin) OR (Rutoside) OR (marina) OR (Kaempferol) OR (kaempferol3) OR (Myricetin) OR (Epicatechin) OR (Catechin) OR (Isorhamnetin) OR (Fisetin) OR (3,3',4',7-Tetrahydroxy-flavone) OR (Chrysin) OR (chrysene) OR (5,7-dihydroxyflavone) OR (Pinocembrin) OR (eriodictyol) OR (abyssinone) OR (Theaflavin) OR (Genistein) OR (Genestein) OR (Daidzein) OR (Theaflavin) OR (Macluraxanthone) OR (Scopoletin) OR (Luteolin) OR (3',4',5,7-Tetrahydroxyflavone) OR (luteolin) OR (Apigenin)  (<https://clarivate.com/webofsciencegroup/solutions/web-of-science-core-collection>)  LIBRARY COCHRANE: 18  (Tooth Wear) OR (Tooth Wears) OR (Wears, Tooth) OR (Wear, Tooth) OR (Dental Wear) OR (Dental Wears) OR (Wear, Dental) OR (Wears, Dental) OR (Enamel wear) OR (Dentin Wear) OR (Erosion, Tooth) OR (Erosive Tooth Wear) OR (Erosive Dentin Wear) OR (Tooth Erosion) OR (Tooth Erosions) OR (Dental Erosion) OR (Dental Erosions) OR (Erosion, Dental) OR (Dental Enamel Erosion) OR (Dental Enamel Erosions) OR (Enamel Erosion, Dental) OR (Erosion, Dental Enamel) OR (Eroded Enamel) OR (Dentin Erosion) OR (Eroded Dentin) OR (Abrasion, Tooth) OR (Abrasion, Dental) OR (Dental Abrasion) OR (Anti-erosive) OR (Protection Erosion) in Title Abstract Keyword AND (Flavonoid) OR (Flavonoids) OR (Bioflavonoids) OR (Bioflavonoid) OR (Flavon) OR (Isoflavones) OR (Flavanones) OR ( Flavanols) OR (Flavanonols) OR (Proanthocyanidin) OR (Naringenin) OR (myricetin) OR (Hesperitin) OR (Hesperidin) OR (Taxifolin) OR (Dihydroquercetin) OR (Quercetin) OR (Quercetine) OR (Dikvertin) OR (Isoquercitrin) OR (isoquercetin) OR (isoquercitin) OR (isotrifoliin) OR (EGCG) OR (Ampelopsin) OR (Rutin) OR (Rutoside) OR (Morina) OR (Kaempferol) OR (Kaempferols) OR (Myricetin) OR (Epicatechin) OR (Catechin) OR (Isorhamnetin) OR (Fisetin) OR (Chrysin) OR (chrysene) OR (Pinocembrin) OR (eriodictyol) OR (Abyssinones) OR (Theaflavin) OR (Genistein) OR (Genestein) OR (Daidzein) OR (Theaflavin) OR (Macluraxanthone) OR (Scopoletin) OR (Luteolin) OR (Luteoline) OR (Apigenin) in Title Abstract Keyword  (<https://www.cochranelibrary.com>) |

## File 2. Characteristics of the scoping sample (n = 34)

| **Domain** | **Category** | **n (%)** |
| --- | --- | --- |
| Study design | In vitro | 20 (58.8) |
|  | In situ | 5 (14.7) |
|  | In vitro + in situ | 7 (20.6) |
|  | Randomized clinical trial | 2 (5.9) |
| Dental substrate | Dentin | 27 (79.4) |
|  | Enamel | 7 (20.6) |
| Experimental condition | Erosion only | 26 (76.5) |
|  | Erosion + abrasion | 7 (20.6) |
|  | Abfraction context | 1 (2.9) |
| Intervention purpose | Therapeutic (on eroded substrates) | 21 (61.8) |
|  | Preventive (on sound tissues) | 13 (38.2) |
| Flavonoid tested | Epigallocatechin-3-gallate (EGCG) | 20 (58.8) |
|  | Proanthocyanidin (PA) | 10 (29.4) |
|  | Quercetin | 5 (14.7) |
|  | Theaflavin | 1 (2.9) |
|  | Hesperidin | 1 (2.9) |
| Formulation / delivery | Solution / rinse | 20 (58.8) |
|  | Gel | 12 (35.3) |
|  | Dentifrice | 2 (5.9) |
|  | Additive (e.g., acid or adhesive) | 1 (2.9) |
| Positive controls | Chlorhexidine (0.012–2 %) | 15 (44.1) |
|  | Sodium fluoride (1000 ppm – 1.23 %) | 12 (35.3) |
|  | Others (SnF₂, KNO₃) | 3 (8.8) |
| Common outcomes | Profilometry (wear) | 34 (100) |
|  | Microhardness | 18 (52.9) |
|  | SEM / CLSM | 16 (47.1) |
|  | Zymography / ICTP assay | 10 (29.4) |
|  | Bond strength | 9 (26.5) |
| Years of publication | 2010 – 2024 | - |
| Regions represented | Brazil  China  Pakistan  Turkey  United States of America  Germany | 21  9  1  1  1  1 |

## File 3. Typical concentration ranges and main endpoints by flavonoid

| **Flavonoid** | **Concentration range / dose band** | **Principal endpoints assessed** |
| --- | --- | --- |
| EGCG | 10–400 µg/mL; 400 µM; 0.6 % (gels up to ≥ 1 %) | ↓ dentin loss; ↑ tubule occlusion; ↑ bond durability; ↓ Carboxy-terminal telopeptide of type I collagen release |
| Proanthocyanidin | 0.05–5 % (gels); 6.5–10 % (primers / mouthrinses) | Collagen stabilization; ↓ wear; ↑ bond strength |
| Quercetin | 75–300 µg/mL; 0.03 % (± NaF synergy) | MMP inhibition; ↑ collagen cross-linking; ↑ microhardness |
| Theaflavin | 1–8 % | Dose-dependent ↓ wear; ↑ cross-linking; ↓ MMP activity |
| Hesperidin | 0.1–1 % | ↓ wear; ↑ tubule occlusion (concentration-dependent) |

**File 4.** Preferred Reporting Items for Systematic reviews and Meta-Analyses extension for Scoping Reviews (PRISMA-ScR) Checklist

| **SECTION** | **ITEM** | **PRISMA-ScR CHECKLIST ITEM** | **REPORTED ON PAGE #** |
| --- | --- | --- | --- |
| **TITLE** | | | |
| Title | 1 | Identify the report as a scoping review. | Page 1.  Title page. |
| **ABSTRACT** | | | |
| Structured summary | 2 | Provide a structured summary that includes (as applicable): background, objectives, eligibility criteria, sources of evidence, charting methods, results, and conclusions that relate to the review questions and objectives. | Page 2.  Abstract page. |
| **INTRODUCTION** | | | |
| Rationale | 3 | Describe the rationale for the review in the context of what is already known. Explain why the review questions/objectives lend themselves to a scoping review approach. | Page 3.  Throughout the  Introduction section. |
| Objectives | 4 | Provide an explicit statement of the questions and objectives being addressed with reference to their key elements (e.g., population or participants, concepts, and context) or other relevant key elements used to conceptualize the review questions and/or objectives. | Page 4.  Introduction  section, last  paragraph. |
| **METHODS** | | | |
| Protocol and registration | 5 | Indicate whether a review protocol exists; state if and where it can be accessed (e.g., a Web address); and if available, provide registration information, including the registration number. | Page 4  (first paragraph). |
| Eligibility criteria | 6 | Specify characteristics of the sources of evidence used as eligibility criteria (e.g., years considered, language, and publication status), and provide a rationale. | Page 4 (paragraph 5) and page 5 (paragraph 1 and 2). |
| Information sources* | 7 | Describe all information sources in the search (e.g., databases with dates of coverage and contact with authors to identify additional sources), as well as the date the most recent search was executed. | Page 5.  Methods section; paragraph 3. |
| Search | 8 | Present the full electronic search strategy for at least 1 database, including any limits used, such that it could be repeated. | Page 5.  (paragraph 3) and  Supplementary  File 1. |
| Selection of sources of evidence† | 9 | State the process for selecting sources of evidence (i.e., screening and eligibility) included in the scoping review. | Page 5.  Paragraphs 4 and 5. |
| Data charting process‡ | 10 | Describe the methods of charting data from the included sources of evidence (e.g., calibrated forms or forms that have been tested by the team before their use, and whether data charting was done independently or in duplicate) and any processes for obtaining and confirming data from investigators. | Page 6  (paragraph 2). |
| Data items | 11 | List and define all variables for which data were sought and any assumptions and simplifications made. | Page 6.  Methods section paragraphs 7 and 8. |
| Critical appraisal of individual sources of evidence§ | 12 | If done, provide a rationale for conducting a critical appraisal of included sources of evidence; describe the methods used and how this information was used in any data synthesis (if appropriate). | Page 6  (paragraph 2). |
| Synthesis of results | 13 | Describe the methods of handling and summarizing the data that were charted. | Page 6  (paragraphs 1 and 2). |
| **RESULTS** | | | |
| Selection of sources of evidence | 14 | Give numbers of sources of evidence screened, assessed for eligibility, and included in the review, with reasons for exclusions at each stage, ideally using a flow diagram. | Page 6  (first paragraph) |
| Characteristics of sources of evidence | 15 | For each source of evidence, present characteristics for which data were charted and provide the citations. | Page 6 and 7  (Paragraphs 2-9.) |
| Critical appraisal within sources of evidence | 16 | If done, present data on critical appraisal of included sources of evidence (see item 12). | Page 6-10.  Bias risk - No applicable |
| Results of individual sources of evidence | 17 | For each included source of evidence, present the relevant data that were charted that relate to the review questions and objectives. | Page 7-10.  Results section paragraphs 10-23. |
| Synthesis of results | 18 | Summarize and/or present the charting results as they relate to the review questions and objectives. | Qualitative Synthesis (Page 6-10);  No metanalysis  performed. |
| **DISCUSSION** | | | |
| Summary of evidence | 19 | Summarize the main results (including an overview of concepts, themes, and types of evidence available), link to the review questions and objectives, and consider the relevance to key groups. | Page 10.  Discussion section paragraphs 2-8. |
| Limitations | 20 | Discuss the limitations of the scoping review process. | Page 14 (last paragraph) and Page 15 (first paragraph). |
| Conclusions | 21 | Provide a general interpretation of the results with respect to the review questions and objectives, as well as potential implications and/or next steps. | Page 14.  Discussion section Last paragraph. |
| **FUNDING** | | | |
| Funding | 22 | Describe sources of funding for the included sources of evidence, as well as sources of funding for the scoping review. Describe the role of the funders of the scoping review. | Page 16 |
